# Supplementary figures and images for: Discovery of RSV-Induced BRD4 Protein Interactions Using Native Immunoprecipitation and Parallel Accumulation—Serial Fragmentation (PASEF) Mass Spectrometry
Source: Viruses. 2021 Mar 11;13(3):454. doi: 10.3390/v13030454 (PMC8000986; doi:10.3390/v13030454)

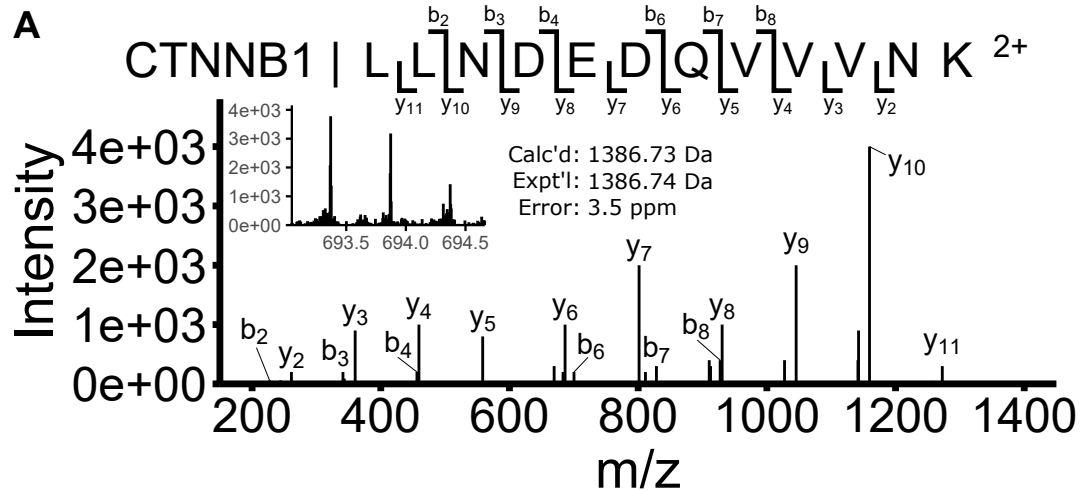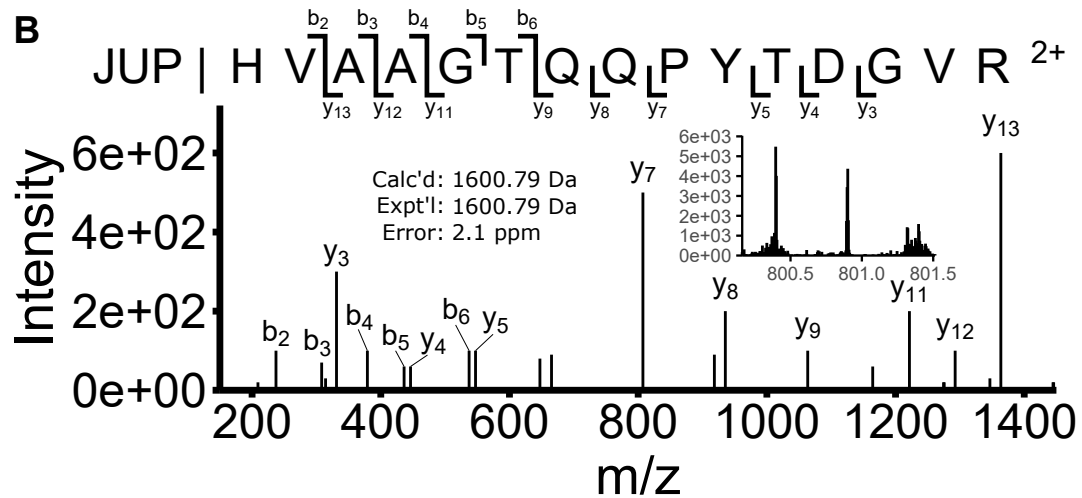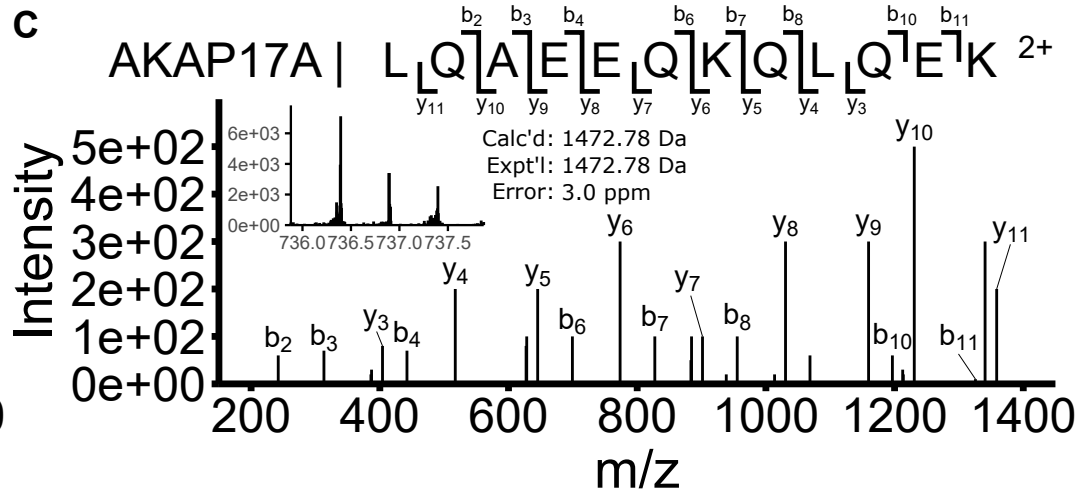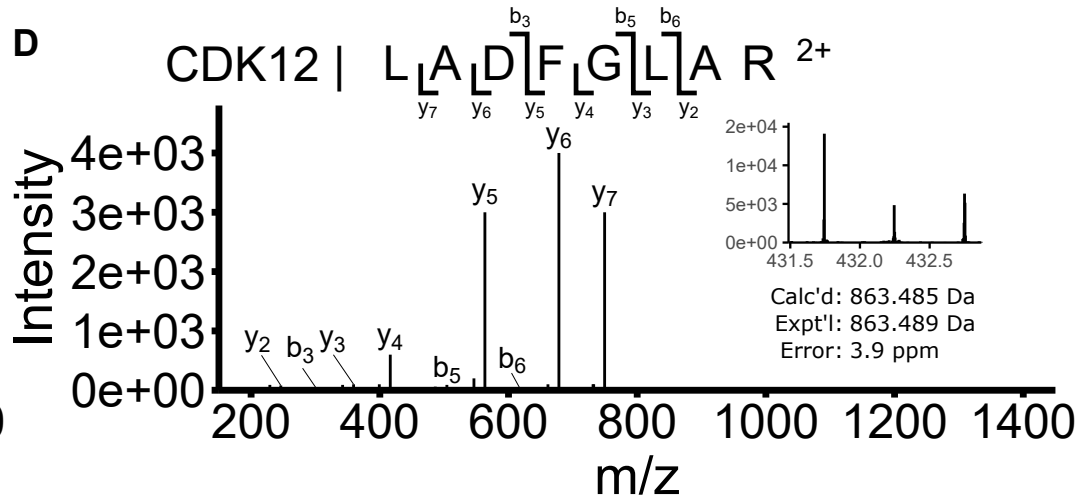

Supplement: Supplementary file 1 [file viruses-13-00454-s001.zip › SI/SI_MS2.pdf]

**A**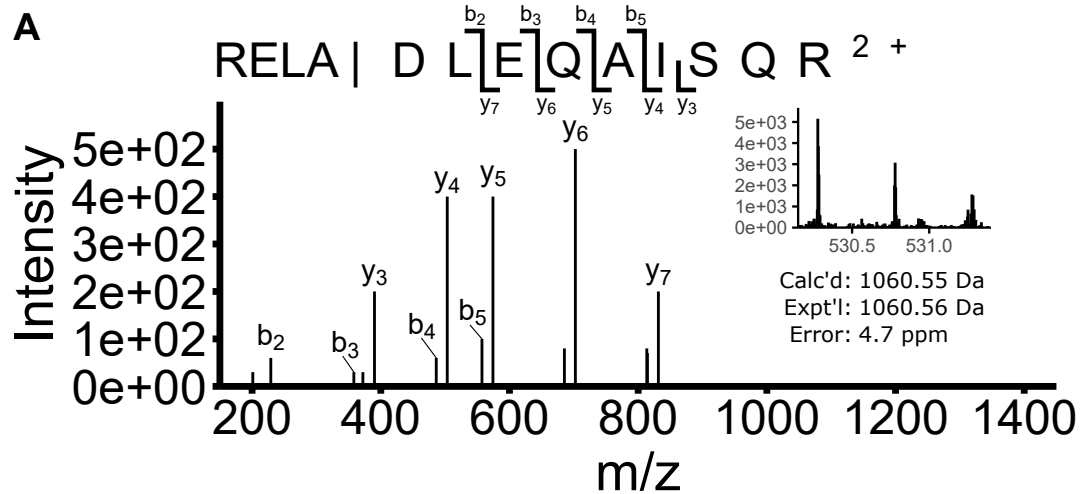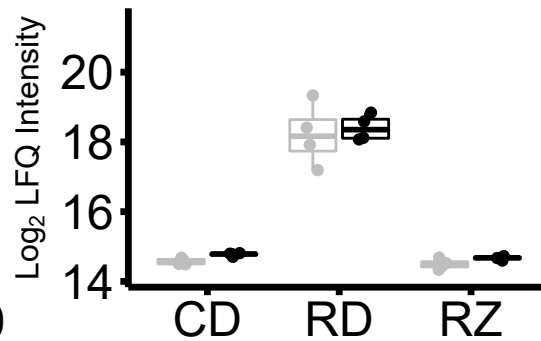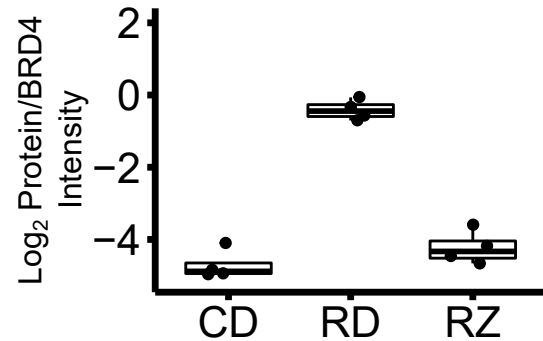**B**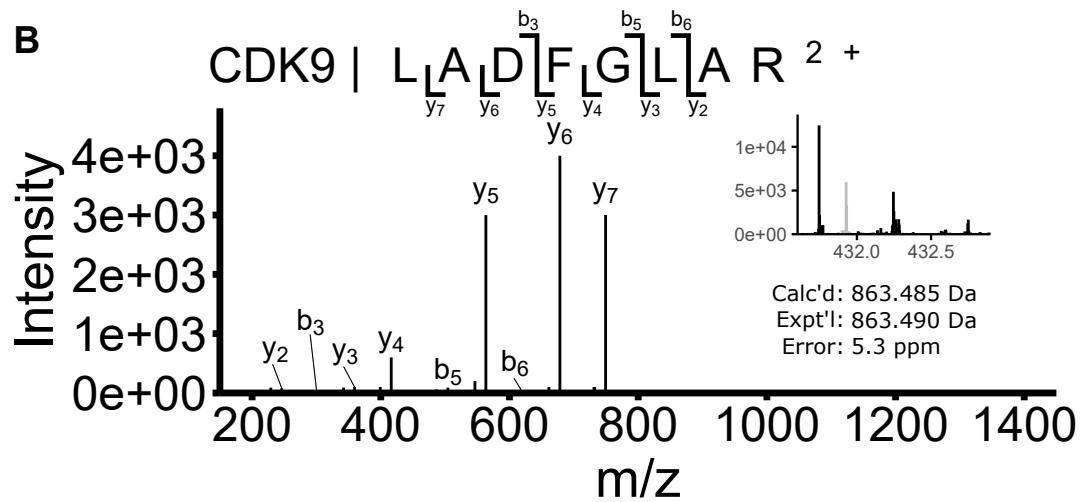

Supplement: Supplementary file 1 [file viruses-13-00454-s001.zip › SI/SI_RELA_CDK9.pdf]

**A**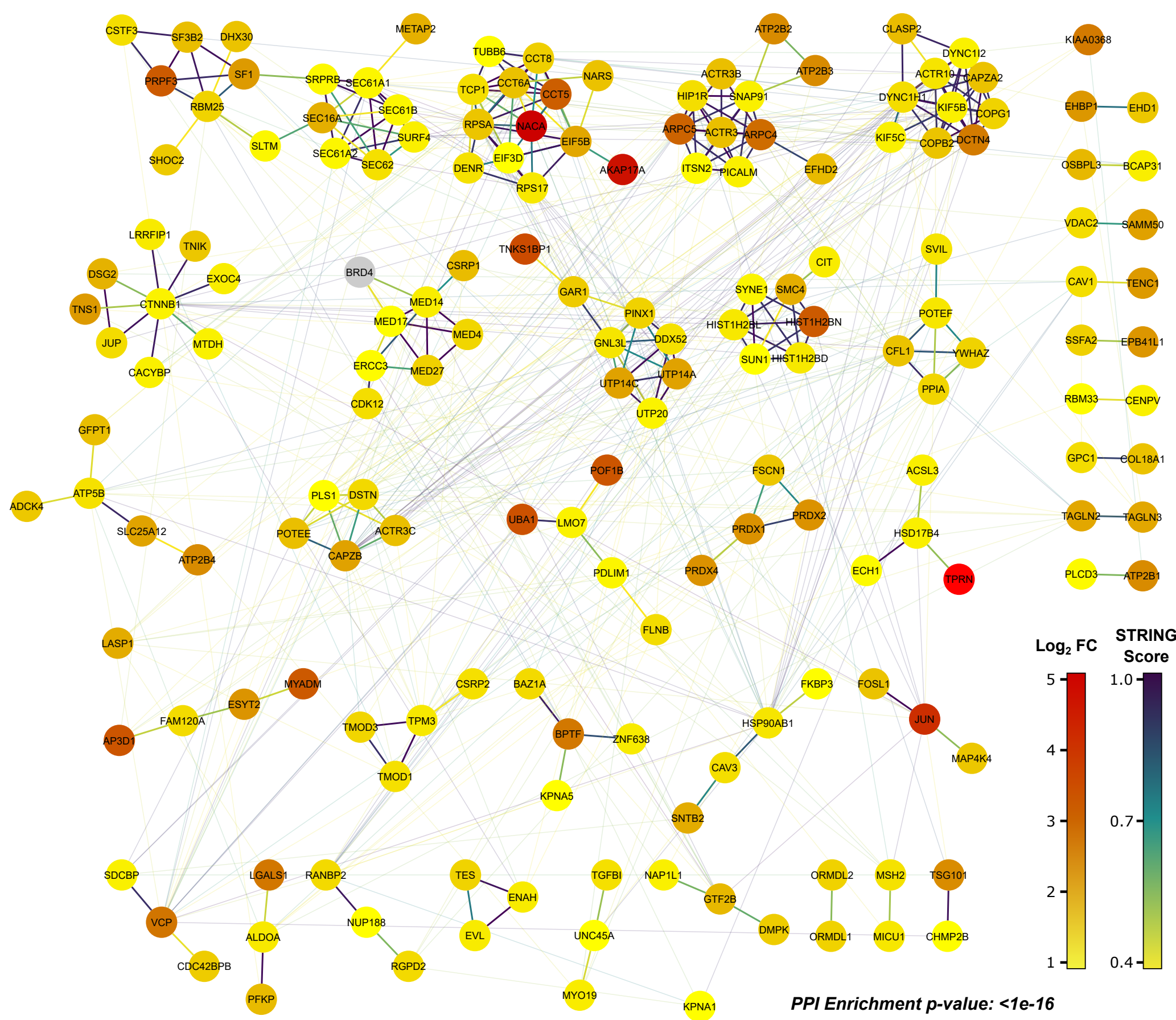**B**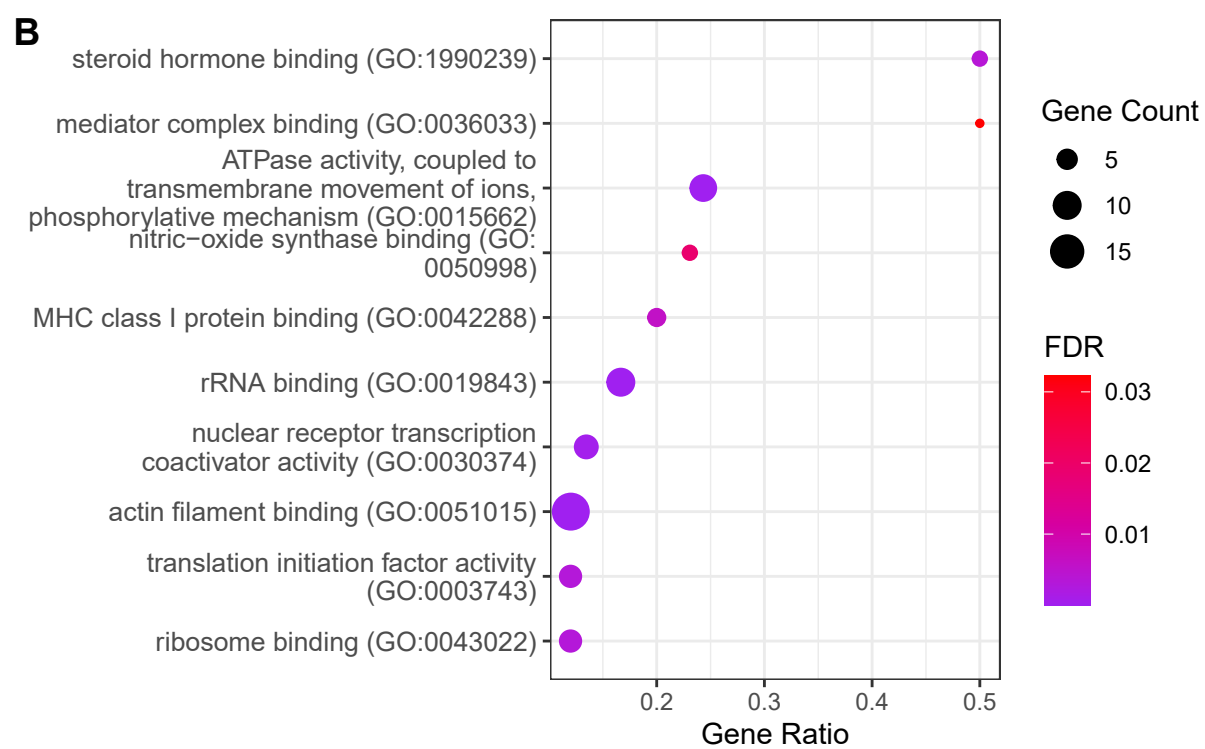**C**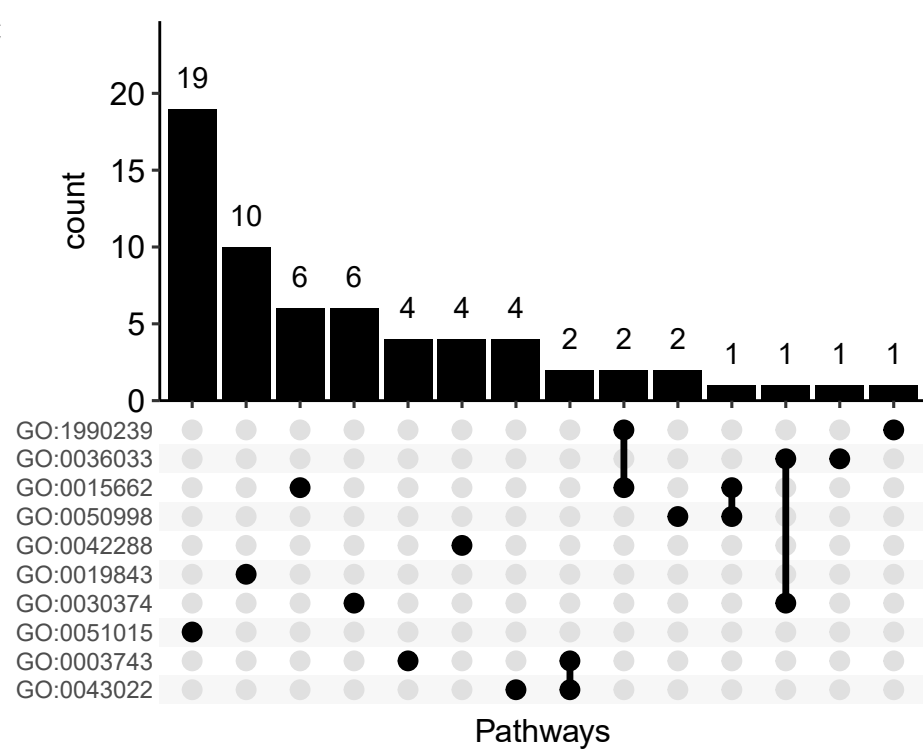

Supplement: Supplementary file 1 [file viruses-13-00454-s001.zip › SI/SI_RSV.pdf]

**A**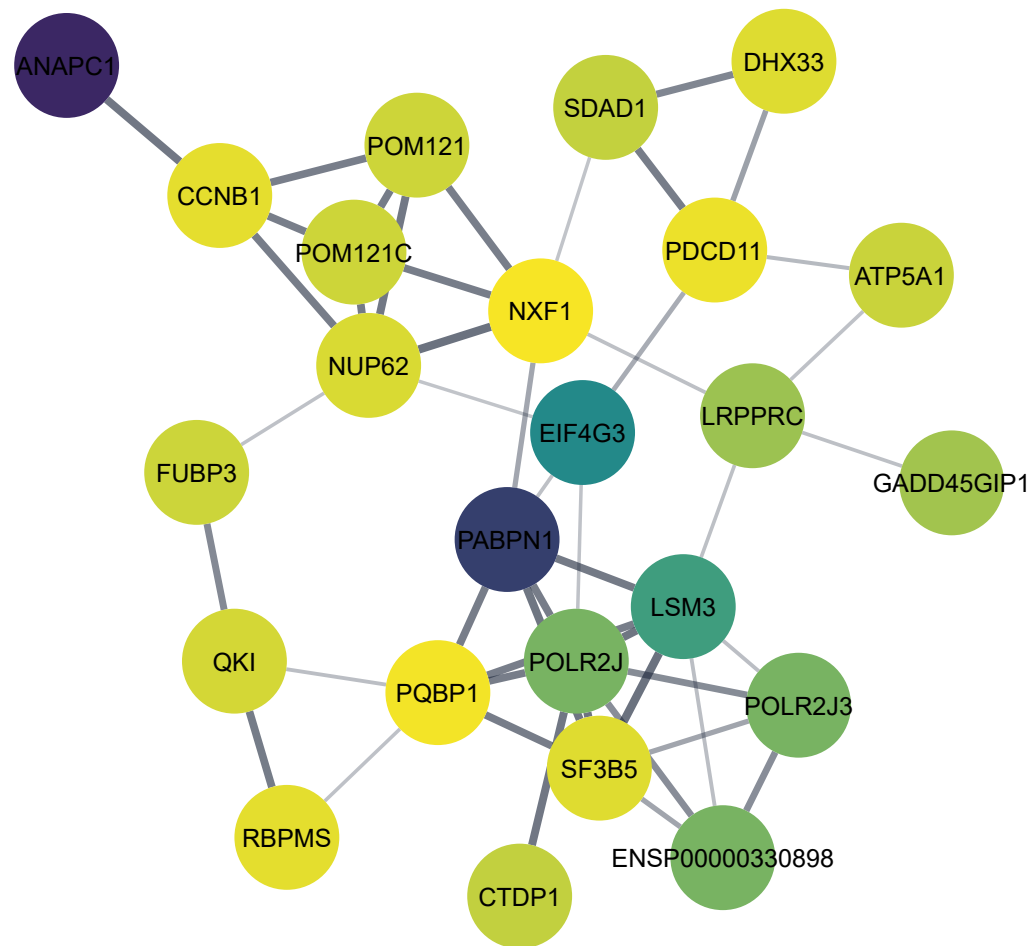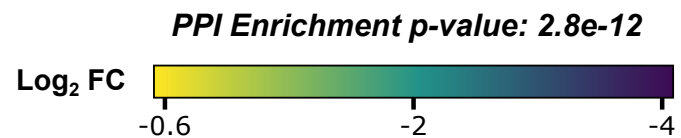**B**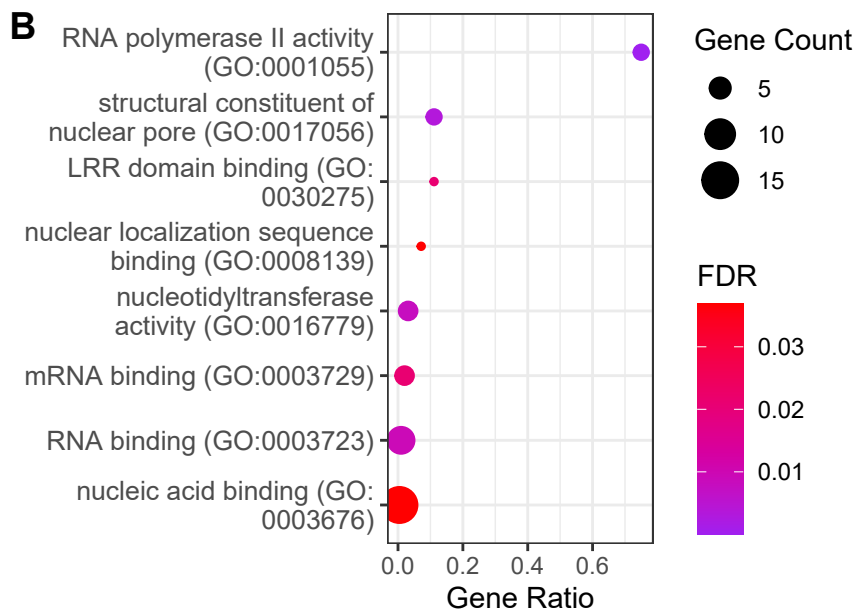**C**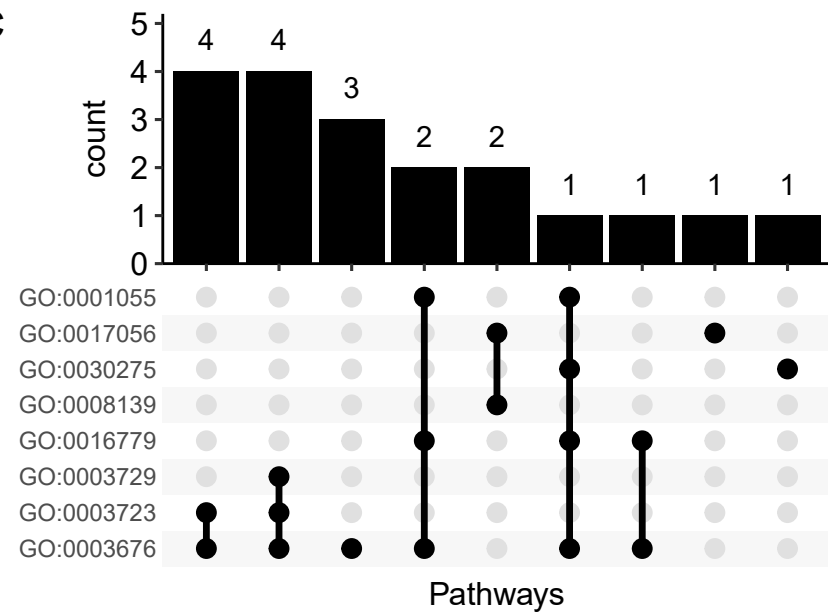

Supplement: Supplementary file 1 [file viruses-13-00454-s001.zip › SI/SI_RSVdown.pdf]
